# Unique Development in Hemichordates Suggests Some Unique Features of Chordates

**DOI:** 10.1371/journal.pbio.0040288

**Published:** 2006-08-22

**Authors:** Richard Robinson

Underlying all the rich variety of form among chordates, from snakes to humans, are several invariant characteristics in body plan. One of the most fundamental of these is the front-to-back, or dorsal–ventral, axis. Our nerve chords run dorsally; our mouths project ventrally. This three-dimensional pattern in the adult is created by a four-dimensional pattern of gene expression during development, as transcription factors turn on and turn off suites of genes in concert.

Many of these transcription factors are even more ancient than the origin of our body plan, and are shared with other creatures, including arthropods, which also have bilateral symmetry and a central nervous system. In a new study, Christopher Lowe, John Gerhart, Marc Kirschner and colleagues show that many of these same signals are employed by the hemichordates, which are the phylum of bilaterally symmetrical adults closest to chordates but surprisingly do not have a central nervous system. However, the developing hemichordate interprets these signals in some ways that are significantly different both from chordates, which they are more closely related to, and arthropods, with which they nonetheless share some important features.

In both chordates and *Drosophila*, the canonical arthropod of the world of research, the dorsal–ventral axis develops in response to opposing gradients of two sets of proteins, Chordin and Bmp. In the embryo, where Chordin is high and Bmp is low, the nervous system develops (on the dorsal side for chordates; on the ventral side for arthropods). Nervous system development proceeds in two phases, both in response to Bmp gradients. First, the ectoderm (one of the three basic tissue layers in the embryo) segregates into epidermis (high Bmp) and neural tissue (low Bmp). Then, within the neural tissue, regions of high Bmp give rise to sensory neurons, while areas of low Bmp give rise to motor neurons and interneurons. (Bmp gradients also influence development of other organ systems in the other tissue layers.)

The acorn worm, *Saccoglossus kowalevskii*, is a hemichordate that lives in intertidal zones and grows to about 8 inches long. It is dorsoventrally polarized in the development and location of its organs, such as the gill slits, the gonads, and the heart/kidney complex. It has a nerve net and axon tracts, but no central nervous system there is no brain-like mass of neural ganglia and, unlike chordates and arthropods, its nerve cells and epidermal cells are finely intermixed.

The authors showed that Bmp and Chordin act within the hemichordate embryo to establish the dorsal–ventral axis, and they identified multiple genes along this axis whose expression was influenced by these two proteins. When they supplied an excess of Bmp, embryos became excessively dorsalized in their expression domains and anatomical features; the opposite occurred when Bmp was diminished. In this respect, the pattern was similar to arthropods and chordates. (Along with its theoretical importance, the identification of this molecular determinant of anatomy aids the practical study of the creature as well, since its anatomy, plus the fact that it spends most of its time in the vertical position, has made it difficult to unambiguously identify a dorsal or ventral side.)

**Figure:**
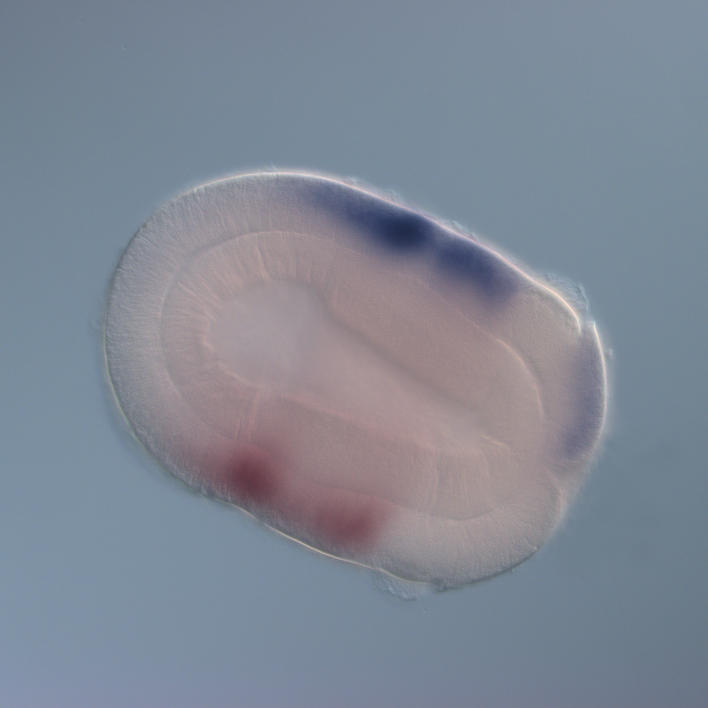
The embryo of the hemichordate *Saccoglossus kowalevskii* expresses the *chordin* gene on the ventral midline and the *bmp2/4* gene on the dorsal midline. All dorsoventral development depends on this Bmp–Chordin polarity.

Unlike in either chordates or *Drosophila*, the first phase of neurogenesis in the hemichordate embryo did not respond to changes in Bmp–Chordin concentration there was no alteration in the neural ectoderm-versus-epidermis differentiation as Bmp was increased. These results suggest that the Bmp–Chordin-mediated centralization of the nervous system, occurring in both arthropods and chordates, arose independently in the two groups after they diverged. The Bmp–Chordin gradient did affect differentiation of neuronal cell types in the hemichordate embryo, analogous to the second phase of patterning in chordates and *Drosophila*, although the details differed significantly. These differences suggest that much of the “regulatory architecture” of the developing nervous system evolved after the chordate–hemichordate split. The full implications of these differences remain to be worked out.

Finally, the results from this study shed light on an important question in comparative evolutionary anatomy. While high Chordin and low Bmp characterize the dorsal side of the chordate embryo, this pattern is associated with the ventral (mouth) side of the *Drosophila* embryo, as well as in all other bilaterally symmetric animals, including the hemichordates. This suggests that during early chordate evolution there was an inversion in the Bmp–Chordin developmental axis relative to the mouth, by movement of the mouth or axis, resulting in the chordate's unique pattern of response to these ubiquitous molecular determinants. Before or after this inversion, the nervous system was centralized to the Chordin (low Bmp) side in the chordate lineage. Further study is needed to answer these questions.

